# Mechanisms of Smartphone Apps for Cigarette Smoking Cessation: Results of a Serial Mediation Model From the iCanQuit Randomized Trial

**DOI:** 10.2196/32847

**Published:** 2021-11-09

**Authors:** Jonathan B Bricker, Michael Levin, Raimo Lappalainen, Kristin Mull, Brianna Sullivan, Margarita Santiago-Torres

**Affiliations:** 1 Division of Public Health Sciences Fred Hutchinson Cancer Research Center Seattle, WA United States; 2 Department of Psychology University of Washington Seattle, WA United States; 3 Department of Psychology Utah State University Logan, UT United States; 4 Department of Psychology University of Jyvaskyla Jyvaskyla Finland

**Keywords:** mediation, engagement, digital, mHealth: smartphone, acceptance, smoking, cessation, app, randomized controlled trial, model, intervention

## Abstract

**Background:**

Engagement with digital interventions is a well-known predictor of treatment outcomes, but this knowledge has had limited actionable value. Instead, learning why engagement with digital interventions impact treatment outcomes can lead to targeted improvements in their efficacy.

**Objective:**

This study aimed to test a serial mediation model of an Acceptance and Commitment Therapy (ACT) smartphone intervention for smoking cessation.

**Methods:**

In this randomized controlled trial, participants (N=2415) from 50 US states were assigned to the ACT-based smartphone intervention (iCanQuit) or comparison smartphone intervention (QuitGuide). Their engagement with the apps (primary measure: number of logins) was measured during the first 3 months, ACT processes were measured at baseline and 3 months (acceptance of internal cues to smoke, valued living), and smoking cessation was measured at 12 months with 87% follow-up retention.

**Results:**

There was a significant serial mediation effect of iCanQuit on smoking cessation through multiple indicators of intervention engagement (ie, total number of logins, total number of minutes used, and total number of unique days of use) and in turn through increases in mean acceptance of internal cues to smoke from baseline to 3 months. Analyses of the acceptance subscales showed that the mediation was through acceptance of physical sensations and emotions, but not acceptance of thoughts. There was no evidence that the effect of the iCanQuit intervention was mediated through changes in valued living.

**Conclusions:**

In this first study of serial mediators underlying the efficacy of smartphone apps for smoking cessation, our results suggest the effect of the iCanQuit ACT-based smartphone app on smoking cessation was mediated through multiple indicators of engagement and in turn through increases in the acceptance of physical sensations and emotions that cue smoking.

**Trial Registration:**

Clinical Trials.gov NCT02724462; https://clinicaltrials.gov/ct2/show/NCT02724462

## Introduction

Cigarette smoking is a leading cause of premature death and disability [1], attributable to over 1 in 10 deaths worldwide [2]. Barriers to accessing evidence-based smoking cessation treatments include low reimbursement for providers and low demand for in-person treatment [3]. Smartphone apps for smoking cessation have been addressing access barriers by serving as digital interventions with high population-level reach [4]. In the United States, the reach of smartphone apps for smoking cessation has been aided by the fact that as of 2019, 81% of all adults owned smartphones—up from 35% in 2011 [5].

Despite their high population-level reach, very little is known about the potential mediators underlying the efficacy of smartphone apps for smoking cessation [6]. In the broader literature on digital interventions (eg, websites and SMS text messaging) for smoking cessation, we are aware of only 3 randomized controlled trials (RCTs) that reported on their mechanisms of action—with each showing support for the theoretical models guiding their interventions (eg, self-efficacy) [7-9]. Understanding mediators is critical for making future improvements to and guiding optimizations of these behavioral interventions [10]. Intervention components that target specific mechanisms of action can be enhanced, with the goal of creating cost-effective changes to increase intervention efficacy, thereby increasing overall impact. Mediational analysis provides a method to identify potential causal links through which the intervention is efficacious [11].

We recently developed and tested iCanQuit, an Acceptance and Commitment Therapy (ACT)–based smartphone app for smoking cessation [12]. In a large 2-arm RCT, iCanQuit was compared to QuitGuide, a US Clinical Practice Guidelines (USCPG)–based smartphone app. At the 12-month follow-up, iCanQuit was 1.5 times more efficacious than QuitGuide for smoking cessation among 2415 smokers (36% racial/ethnic minority groups) from all 50 US states [12]. The importance of the iCanQuit study is that it is the first full-scale RCT with long-term follow-up to show that a smartphone app was efficacious for smoking cessation [4].

It remains unknown why iCanQuit was efficacious. The iCanQuit intervention targeted 2 core processes of ACT [13]: acceptance and values. Specifically, ACT teaches acceptance of internal cues to smoke (sensations, emotions, and thoughts), which is conceptually distinct from USCPG-based standard approaches that teach avoidance of internal cues to smoke [14,15]. ACT also motivates smokers to quit by appealing to their values, whereas the USCPG-based approaches motivate through reason and logic [14,15]. The iCanQuit app was designed to change the level of enactment of personal values through exercises focusing on valued life domains inspiring a user to quit smoking (eg, family, health, and spirituality) and planning weekly actions to take in those life domains (eg, going on a walk with one’s partner).

Acceptance has been identified as a core mediator in ACT-based interventions across a wide variety of content areas [16-18]. For smoking cessation interventions, prior studies have shown that acceptance of internal cues to smoke was a mediator of intervention efficacy [14,19]. For example, we found that in the WebQuit trial of an ACT-based website intervention for smoking cessation, baseline to 3-month increases in acceptance accounted for 80% (*P*<.001) of the effect of WebQuit.org on the main cessation outcome [14]. There is also evidence indicating that the enactment of values mediates the effects of ACT when applied to various mental health and chronic health conditions [20-23]. These mediation findings are consistent with ACT theory and treatment protocols, including the iCanQuit program, in which there is a strong emphasis on values in addition to acceptance. However, to date, the mediational role of enacting one’s values as a way to motivate smokers to quit smoking has not been empirically tested in smoking cessation interventions.

In parallel with studies on psychological mechanisms of action, digital intervention researchers have been studying the role of intervention engagement as a process that predicts treatment outcomes [6,7,24,25]. Our previous study has shown that, in the SmartQuit app that preceded the iCanQuit app, engagement with the intervention and its specific ACT components was predictive of smoking cessation. Participants who completed the program were over 4 times more likely to quit smoking. This app had a tool to track when a user “let a craving pass,” defined as noticing a craving and not acting on it by smoking. Usage of this tracking tool predicted a greater likelihood of quitting smoking [26]. Building on this research, the next step is to learn why engagement predicts cessation. By itself, engagement is a limited explanatory variable: engagement describes the user’s actions; however, it is unclear how those actions lead to successful treatment outcomes [27].

**Figure 1:**
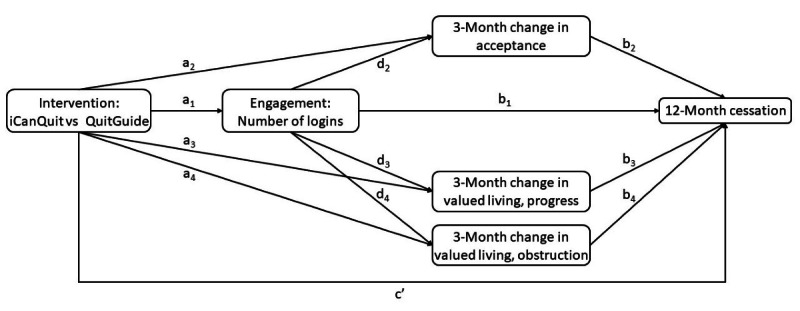
Conceptual model for serial mediation of potential mediators of the iCanQuit intervention.

As shown in Figure 1, we posit that the effect of the intervention (iCanQuit vs QuitGuide) on smoking cessation at the 12-month follow-up may be mediated by engagement (number of logins), which, in turn, impacts 3-month changes in acceptance and valued living. Specifically, the appeal and utility of iCanQuit’s content (eg, ACT skills modules) may contribute to higher user engagement as compared to the QuitGuide intervention. This higher engagement may lead to changes in the 2 ACT-based processes targeted in the iCanQuit intervention: (1) higher levels of acceptance of internal smoking cues and (2) enactment of one’s values as measured by progress and obstruction of valued living, respectively. Both acceptance of internal cues to smoke and enactment of one’s values may then lead to a higher likelihood of quitting smoking. Therefore, this study aimed to test this serial mediational model in the full-scale iCanQuit trial. These results will provide the first known evidence on potential serial mediators of smartphone apps for smoking cessation. While such serial mediational models are useful for developing an in-depth understanding of intervention efficacy, they are rare in smoking cessation research [28-30].

## Methods

### Design

Data for this secondary analysis were obtained from all 2415 individuals enrolled in the 2-arm iCanQuit RCT for smoking cessation, with its complete details previously described [12]. In brief, a racially and ethnically diverse sample of 2415 adult daily smokers from all 50 US states were randomized 1:1 to either receive access to an ACT-based smartphone app (iCanQuit) or a USCPG-based smartphone app (QuitGuide) for smoking cessation.

### Eligibility Criteria

Eligibility criteria included: (1) being 18 years of age or older, (2) having smoked 5 or more cigarettes per day in the past year, (3) wanting to quit smoking within the subsequent 30 days, (4) if concurrently using any other tobacco products, wanting to quit consuming all tobacco products within 30 days, (5) having an interest in learning skills to quit smoking and being willing to be randomized to either treatment condition, (6) having daily access to their own smartphone, (7) knowing how to download smartphone apps, (8) being willing and able to read in English, (9) having never used QuitGuide and not currently using another smoking cessation treatment, (10) having never participated in our prior studies, (11) no household members having been already enrolled, (12) being willing to complete outcome surveys, and (13) being able to provide contact information for themselves and 2 relatives.

### Recruitment, Enrollment, and Follow-up

Adults were recruited nationwide via Facebook ads, a survey sampling company, search engine results, and friends/family referral. Participants completed an encrypted, web-based screening survey and were notified of their eligibility via email. They then clicked on their secured emailed link to the study website, where they provided consent and completed the baseline survey. At each enrollment step, the study was presented as a comparison of 2 smartphone apps for smoking cessation.

Participants were randomized (1:1) to either iCanQuit or QuitGuide using randomly permuted blocks of size 2, 4, and 6, stratified by daily smoking frequency (≤20 vs ≥21), education (≤high school vs ≥some college), race/ethnicity (minority race/ethnicity vs non-Hispanic White), and positive screening for depression (CES-D score ≤15 vs ≥16) [31]. Random assignments were concealed from participants throughout the trial. The random allocation sequence was generated by a database manager and implemented automatically by the study website. Neither research staff nor study participants had access to upcoming randomized assignment into either study arm. In both arms, participants could access their interventions from the moment of randomization and beyond (ie, after the end of the 12-month follow-up period). All participants provided consent online and were compensated to up to US $105 for completing study data collection. All study activities were approved by the institutional review board of the Fred Hutchinson Cancer Research Center.

### Interventions

#### iCanQuit

Participants randomized to the iCanQuit arm received access to download the iCanQuit smartphone app (version 1.2.1). iCanQuit intervenes on the ACT-focused processes of acceptance of internal cues to smoke and enactment of one’s values that guide quitting smoking [12]. The acceptance component of the app teaches skills to accept physical sensations, emotions, and thoughts that trigger smoking by distancing from thoughts about smoking, mindfulness skills, and flexible perspective-taking. The values component of the app teaches skills for determining the core life domains that motivate quitting smoking (eg, family, health, and spirituality) and taking repeated small actions within these domains (eg, playing with grandchildren) to develop a smoke-free life. The program is self-paced, and the content is unlocked in a sequential manner across 8 levels. Each of the first 4 levels is made accessible immediately after the prior level is completed, while each of the last 4 is only unlocked upon recording 7 consecutive days without smoking. If a participant lapses, the program encourages (but does not require) them to set a new quit date and return to the first 4 levels for preparation. The program also includes on-demand tools to help in coping with smoking urges and to track the daily number of cigarettes smoked and urges passed without smoking.

#### QuitGuide

Participants randomized to the QuitGuide arm received access to download the QuitGuide smartphone app (version 1.2.2). QuitGuide content is delivered in four main sections: (1) “Thinking about quitting,” which focuses on motivations to quit by using reason and logic such as identifying reasons to quit and providing information on the health consequences of smoking and quitting; (2) “Preparing to Quit,” which helps users develop a plan to quit, identify smoking behaviors, triggers, and reasons for being smoke-free, and social support for quitting; (3) “Quitting,” which teaches skills for avoiding cravings to smoke; and (4) “Staying Quit,” which presents tips, motivations, and actions to stay smoke-free and skills for coping with slips. No quit smoking medications, coaching, or any other intervention was provided in either intervention arm [12].

### Study Measures

#### Baseline Characteristics and Covariates

Data collected at baseline included age, gender, ethnicity, education, employment, income, marital status, and sexual orientation. Study participants completed validated positive screening tools to assess mental health, including depression [31], panic [32], and posttraumatic stress disorder [33]. Alcohol consumption and heavy drinking were assessed via the Quick Drinking Screen [34]. Smoking behavior variables included nicotine dependence (measured using the Fagerström Test for Nicotine Dependence) [35], number of cigarettes smoked per day, years of smoking, use of e-cigarettes, quit attempts, and relationships with other people who smoke.

As reported in the parent trial paper (and thus not reported in this study), participants were from all 50 US States. The mean age at enrollment was 38.2 (SD 10.9) years. Participants were 70.4% (1700/2415) women and 35.9% (868/2415) reported racial/ethnic minority backgrounds. There were 41.2% (995/2415) with high school or less education. Regarding smoking, 83.1% (2009/2415) had smoked for ≥10 years and 74.7% (1803/2415) smoked more than a half pack (at least 11 cigarettes) per day. There were no significant differences between the 2 arms on any baseline variable (for all, *P*>.05) [12].

#### Treatment Engagement Mediator: Baseline to 3 Months

Engagement with the assigned app was objectively measured using Google Analytics. The main mediational model’s measure of engagement was the number of times each app was opened, consistent with other digital interventions’ measures of engagement [7,24,25]. App activity that occurred at least 10 minutes after previous activity was considered a new login. Secondary measures of engagement were the total number of minutes and the unique number of days on which each app was used. To test the proposed mediational model (Figure 1), the first 3 months of utilization data for each participant were used in this study (N=2415).

#### ACT Theory–Based Mediators: Baseline to 3 Months

Change from baseline to 3 months after randomization in ACT theory–based processes, including acceptance of internal cues to smoke and valued living, were measured using validated tools. Acceptance of internal cues to smoke was measured via the Avoidance and Inflexibility Scale (AIS) [36], using the mean of the three 9-item subscales that assess one’s willingness to experience physical sensations, emotions, and thoughts that cue smoking. The items are rated on a 5-point scale from (1) “Not at all” to (5) “Very willing” and averaged, with higher scores indicating greater acceptance. A sample physical sensation item was “How willing are you to notice these bodily sensations without smoking?” and items from the emotions and thoughts subscales were similar, substituting “feelings” or “thoughts” for “bodily sensations.” Valued living was measured using the 10-item Valuing Questionnaire (VQ) [37] designed to assess the extent of enactment of personal values. Each item is rated on a 7-point scale ranging from (0) “Not at all true” to (6) “Completely true.” Scores were averaged and 2 distinct factors were derived, progress and obstruction, with higher scores indicating either greater progress or greater obstruction toward valued living, respectively. A sample progress item was “I worked toward my goals even if I didn’t feel motivated to” and a sample obstruction item was “I was basically on auto-pilot most of the time.” Cronbach α (95% CI) values for each of the 3 scales showed good internal consistency: (1) mean acceptance [Cronbach α=.76 (95% CI .75-.77)], (2) valued living, progress subscale [Cronbach α=.88 (95% CI .87-.89)], and (3) valued living, obstruction subscale [Cronbach α=.88 (95% CI .87-.89)].

#### Smoking Cessation Outcome: 12 Months

The parent trial’s primary smoking cessation outcome was specified a priori as self-reported complete-case 30-day point-prevalence abstinence (PPA) at the 12-month follow-up. The secondary smoking cessation outcome for this study was intent-to-treat missing as smoking 30-day PPA at the 12-month follow-up. As reported in the parent trial, for the primary outcome of 30-day PPA at the 12-month follow-up, iCanQuit participants had a 1.49-fold higher odds of quitting smoking as compared to QuitGuide participants (28.2%, 293/1040 abstinent vs 21.1%, 225/1067 abstinent; odds ratio [OR] 1.49, 95% CI 1.22-1.83; *P*<.001). When missing data were coded as smokers, 12-month 30-day PPA results were very similar: 24.1% (293/1214) abstinent for iCanQuit vs 18.7% (225/1201) abstinent for QuitGuide (OR 1.40, 95% CI 1.14-1.71, *P*<.001).

### Statistical Analyses

We first compared treatment arms on proposed mediators at 3 months, using a negative binomial model for the number of logins owing to its highly right-skewed distribution and generalized linear models for the remaining mediators (ie, change in mean acceptance, and valued living progress and obstruction subscales). Regression analyses were performed using R (version 4.0.3, The R Foundation) [38] and the “MASS” library for negative binomial regression [39]. Hayes’ PROCESS macro (version 3.5) for SAS [40] was used to test serial mediation of the effect of intervention condition on cessation at 12 months through engagement and through changes in acceptance and valued living from baseline to 3 months. Using the notation in Figure 1, the indirect effect of the intervention on cessation through the number of logins alone was estimated by *a*_1_*b*_1_. Similarly, the indirect effects through change in acceptance and valued living progress and obstruction subscales were estimated by *a*_2_*b*_2_, *a*_3_*b*_3_, and *a*_4_*b*_4_, respectively. The serial mediation effects determined through the number of logins and in turn through change in acceptance and valued living progress and obstruction subscales were estimated by *a*_1_*d*_2_*b*_2_, *a*_1_*d*_3_*b*_3_, and *a*_1_*d*_4_*b*_4_, respectively. Indirect effects were estimated with 5000 bootstrapped samples and were considered statistically significant when 95% CIs did not include zero. Model covariates included the 4 factors used in stratified randomization (ie, education level, heavy smoking [≥21 cigarettes per day], minority race or ethnicity, depression symptoms [20-item Center for Epidemiological Studies-Depression scale score ≥16], and baseline acceptance and valued living scores). This approach, in which the analysis is consistent with the stratified randomization study design, has been recommended to avoid losing power and obtaining incorrect 95% CIs [41,42].

Primary analyses were conducted with complete-case data for all variables in the serial mediation model, which was available for 1846 participants. As reported in the parent trial, the follow-up data retention was 86.7% (n=2093/2415) overall at 3 months (85.9%, 1043/1214 for iCanQuit vs 87.4%, 1050/1201 for QuitGuide [*P*=.28] and 87.2% (n=2107/2415) overall at 12 months (85.7%, 1040/1214 for iCanQuit vs 88.8%, 1067/1201 for QuitGuide [*P*=.02]) [12]. A sensitivity analysis for the serial mediation model was performed using full information maximum likelihood to handle missing data in Mplus [43]. Secondary mediation analyses included all 3 AIS acceptance subscales (ie, willingness to experience physical sensations, emotions, and thoughts that cue smoking), and alternative measures of engagement (ie, total time measured as minutes of app use and the number of unique days of use).

## Results

As shown in Table 1, participants randomized to iCanQuit logged into their assigned app for a significantly greater number of times than those randomized to QuitGuide (25.7 vs 7.5 times; *P*<.001). In addition, they had greater baseline to 3-month increases in acceptance of cues to smoke (*P*<.001). However, changes in the valued living subscales of progress and obstruction were not different between the 2 treatment arms (for all, *P*>.05). Table 1 also shows that for every 1-point increase from baseline to 3 months in acceptance of cues to smoke, there was a 6.07-fold higher odds of 12-month smoking cessation (OR 6.07, 95% CI 4.76-7.76, *P*<.001).

**Table 1:** Differences in mediators between the 2 intervention arms at 3-month follow-up and the effect of each 1-point increase in mediator on 12-month cessation outcomes.

	Relationship between treatment arm and mediator (*a* paths)					*P* value		Relationship between mediator and cessation (*b* paths)	
Mediator	Total (n=1846), mean (SD)	QuitGuide (n=929), mean (SD)	iCanQuit (n=917), mean (SD)	Incidence rate ratio or point estimate (95% CI)			Odds ratio (95% CI)		*P* value
Number of logins	16.5 (32.3)	7.5 (14.0)	25.7 (41.6)	3.46^a^ (3.10 to 3.87)	<.001		1.01 (1.01-1.02)		<.001
Change in mean acceptance	0.13 (0.57)	0.06 (0.50)	0.20 (0.62)	0.13^b^ (0.09 to 0.18)	<.001		6.07 (4.76-7.76)		<.001
Change in valued living-progress	–0.67 (7.88)	–0.72 (7.75)	–0.62 (8.01)	–0.12^b^ (–0.74 to 0.50)	.71		1.04 (1.02-1.05)		<.001
Change in valued living-obstruction	0.43 (8.28)	0.51 (7.79)	0.35 (8.75)	0.15^b^ (–0.49 to 0.78)	.65		0.96 (0.95-0.98)		<.001

^a^Incidence rate ratio values.

^b^Point estimate values.

The results of the primary serial mediation model are shown in Table 2 and they show the indirect effects posited by the model rather than individual path coefficients. Baseline to 3-month number of logins (indirect effect *a*_1_*b*_1_=0.09, 95% CI 0.04-0.18, *P*<.001) and change in mean acceptance of internal cues to smoke (indirect effect *a*_2_*b*_2_=0.12, 95% CI 0.04-0.21, *P*<.001) each mediated the effect of intervention condition on smoking cessation at 12 months. There was a significant serial mediation effect of intervention condition on smoking cessation through the number of logins and in turn through the change in mean acceptance (indirect effect *a*_1_*d*_2_*b*_2_=0.11, 95% CI 0.07-0.15, *P*<.001). This serial mediation effect corresponds to an OR of 1.11 (95% CI 1.08-1.16). In contrast, none of the pathways through valued living subscales, neither progress nor obstruction, mediated the relationship between intervention condition and cessation. This pattern of results was the same for the missing as smoking cessation outcome.

**Table 2:** Estimates of indirect effects for all pathways in the serial mediation model.

Mediator	Path	Estimate of indirect effect (95% CI) for complete-case cessation outcome^a^	Estimate of indirect effect (95% CI) for missing as smoking cessation outcome^a^
Number of logins	*a*_1_ *b*_1_	0.09 (0.04 to 0.18)^b^	0.10 (0.05 to 0.18)^b^
Change in mean acceptance	*a*_2_ *b*_2_	0.12 (0.04 to 0.21)^b^	0.12 (0.04 to 0.20)^b^
Change in valued living, progress subscale	*a*_3_ *b*_3_	–0.01 (–0.02 to 0.01)	0.00 (–0.02 to 0.01)
Change in valued living, obstruction subscale	*a*_4_ *b*_4_	0.00 (–0.01 to 0.01)	0.00 (–0.01 to 0.01)
Number of logins and change in mean acceptance, in serial	*a*_1_ *d*_2_ *b*_2_	0.11 (0.07 to 0.15)^b^	0.10 (0.07 to 0.14)^b^
Number of logins and change in valued living progress, in serial	*a*_1_ *d*_3_ *b*_3_	0.00 (–0.001 to 0.01)	0.00 (–0.001 to 0.01)
Number of logins and change in valued living obstruction, in serial	*a*_1_ *d*_4_ *b*_4_	0.00 (–0.003 to 0.002)	0.00 (–0.003 to 0.002)

^a^95% CIs that include 0 are nonsignificant. Indirect effect estimate (95% CI) values may be exponentiated to produce estimates on the odds ratio scale.

^b^*P*<.05.

In secondary analysis models, the pattern of results for the serial mediation model was the same when engagement was measured as the total number of minutes (indirect effect *a*_1_*d*_2_*b*_2_=0.09, 95% CI 0.05-0.14, *P*<.001) or the total number of unique days on which each app was used (indirect effect *a*_1_*d*_2_*b*_2_=0.13, 95% CI 0.10-0.17, *P*<.001). This is consistent with the high correlations between engagement measures, which ranged from 0.72 to 0.91 (results not shown). Results were the same when the mediation model was reanalyzed with full information maximum likelihood (N=2415; data not shown).

The primary mediation model was further elaborated in a sensitivity analysis to determine which acceptance subscales mediated the effect of intervention on smoking cessation at 12 months (Multimedia Appendices 1 and 2). Our results show that change in the mean acceptance of physical sensations (indirect effect *a*_2_*b*_2_=0.03, 95% CI 0.02-0.06, *P*<.001) and acceptance of emotions (indirect effect *a*_4_*b*_4_=0.09, 95% CI 0.03-0.16, *P*<.001), but not acceptance of thoughts (indirect effect *a*_3_*b*_3_=0.01, 95% CI –0.02 to 0.04, *P*>.05), each mediated the effect of the intervention condition on smoking cessation at 12 months. Regarding serial mediation, the effect of the intervention condition on smoking cessation was significantly mediated through the number of logins and in turn through change in the mean acceptance of physical sensations (indirect effect *a*_1_*d*_2_*b*_2_=0.03, 95% CI 0.01-0.05, *P*<.001) and acceptance of emotions (indirect effect *a*_1_*d*_4_*b*_4_=0.07, 95% CI 0.04-0.11, *P*<.001). In contrast, the serial mediation pathway through acceptance of thoughts was not significant (indirect effect *a*_1_*d*_3_*b*_3_=0.01, 95% CI –0.01 to 0.03, *P*>.05). Similar to the primary model, none of the pathways through valued living subscales, neither progress nor obstruction, mediated the relationship between intervention condition and cessation (for all, *P*>.05).

## Discussion

This is the first study of serial mediators underlying the efficacy of smartphone apps for smoking cessation in a nationwide sample of daily smokers. The study tested whether the effect of the iCanQuit (vs QuitGuide) intervention on smoking cessation at the 12-month follow-up was mediated by engagement that in turn impacted 3-month changes in acceptance and valued living. Overall, there was a significant serial mediation effect of iCanQuit on smoking cessation through multiple indicators of engagement (ie, total number of logins, total number of minutes, and total number of unique days or use) and in turn, through change in mean acceptance of internal cues to smoke. Supplementary analysis of the acceptance subscales showed that serial mediation was through acceptance of physical sensations and emotions but not acceptance of thoughts. There was no evidence that the effect of the iCanQuit intervention (vs QuitGuide) was mediated by changes in valued living.

The results significantly advance the understanding of mechanisms underlying interventions for smoking cessation, and digital interventions for smoking cessation in particular. To date, serial mediation models of smoking cessation have been rare. One study found that the effect of telemedicine for smoking cessation on cessation was mediated by providers’ support, which, in turn, led to increased self-efficacy and impacted cessation [28]. Another study found that the effect of financial incentives on quitting smoking was mediated only by self-efficacy but not program satisfaction [30]. The unique value of the current study’s serial mediation model is in demonstrating how treatment engagement leads to higher cessation outcomes [7,24,25]. Our results suggest that regardless of the measure of engagement, greater treatment participation leads to greater improvements in underlying theoretical processes of behavior change, which in this case was the ACT process of acceptance of internal cues to smoke. This provides empirical support to the clinical premise that greater usage of the mobile app is a key pathway to activating a person’s learning of therapeutic processes of change. The serial mediation findings indicate that part of how greater engagement leads to greater likelihood of smoking cessation is through activation of key psychological processes targeted in the intervention. Future research can examine whether engagement in certain types of clinical content (eg, specific behavior change exercises) have a stronger link to mediating certain therapeutic processes than others, which could provide the empirical guidance to further optimize interventions to increase engagement with behavioral intervention components that most effectively target key psychological processes. This knowledge could inform smartphone intervention designs that coherently connect program engagement, program components, and therapeutic processes to improve treatment outcomes.

The results on acceptance have several important implications for the ACT model of smoking cessation. Eight prior ACT RCTs showed either formal statistical mediation or higher levels of acceptance of internal cues to smoke in the ACT intervention arm [14,15,44-49]. Building on this evidence, our results suggest that acceptance of physical sensations (eg, cravings) and emotions that trigger smoking, but not acceptance of thoughts that trigger smoking, may be important theoretical pathways of smoking cessation. These findings contrast with those of the general ACT therapeutic model, in which acceptance of thoughts, and related changes in how one responds to thoughts, is theorized to be an important therapeutic process for ACT and major component of treatment [13]. If replicated, these findings suggest a potential point for theory refinement in applying the ACT model for smoking cessation.

For intervention design, these findings suggest that future digital ACT-focused smoking cessation interventions should emphasize targeting acceptance of cravings and emotions that cue smoking. This could be accomplished by focusing on intervention exercises that help people (1) identify physical sensations and emotions that trigger smoking behaviors and (2) practice openness and willingness to experience these sensations and emotions. Skills-training in allowing cravings to pass and mindful awareness of cravings and emotions may be especially beneficial. In contrast, these findings suggest that less focus should be on exercises targeting acceptance of thoughts that trigger smoking since this does not appear to mediate treatment effects on smoking cessation.

The results on valued living are novel and have implications for future research. To date, no prior research has examined the role of valued living in smoking cessation. In the broader literature on ACT intervention research, we are only aware of a few pilot studies, all among college students, which showed that the effects of ACT digital interventions for stress, anxiety, and depression were mediated by valued living or meaningfulness [22,23]. Our results on valued living suggest some possibilities. Primarily, valued living or enactment of one’s values may not be a mediator of smoking cessation. While it is conceivable that one does not need to work toward broader life goals to quit smoking, this is an unlikely explanation given the central role of motivation in health behavior change overall [50,51]. A more plausible explanation is that the current measure of valued living is not a sensitive measure of valued actions pertinent to smoking cessation. The VQ pertains to one’s overall sense of life purpose and goals, whereas a smoking cessation intervention like iCanQuit focuses specifically on valued life domains directly associated with smoking (eg, health) as motivators to take actions toward quitting (eg, setting a quit date). The measure of acceptance was specific to smoking [36], rather than a general construct of acceptance of internal experience [52] and, as observed in this study, the associations between acceptance of smoking cues and smoking cessation were significant. In contrast, the observed associations between valued living, as measured broadly by the VQ, and smoking cessation were minor. Nonetheless, the predictive relationship between valued living and smoking cessation was significant. Thus, another possibility is that while iCanQuit focuses on values specifically in the context of smoking cessation, there may be some benefits to adding a general, less smoking-specific, intervention for valued living. Finally, it is worth noting that prior research has shown mixed evidence for the sensitivity of the VQ in detecting ACT intervention effects, suggesting that there may be limitations in the scale [22,53,54]. Future research can focus on developing a smoking-specific valuing questionnaire with the ultimate goal of testing it in smoking cessation intervention research.

In conclusion, this is the first study of serial mediators underlying the efficacy of smartphone apps for smoking cessation. The effect of the iCanQuit smartphone app on smoking cessation was mediated through multiple indicators of engagement and, in turn, through change in acceptance of physical sensations and emotions. Our results suggest that smoking cessation interventions should focus on increasing treatment engagement with the goal of enhancing the acceptance of cravings and emotions that cue smoking.

## Appendix

Multimedia Appendix 1 Conceptual model for serial mediation analysis including the three acceptance subscales.

Multimedia Appendix 2 Estimates of indirect effects for pathways in a serial mediation model including the three Avoidance and Inflexibility Scale (AIS) subscales.

Multimedia Appendix 3 CONSORT-eHEALTH checklist (V 1.6.1).

## Abbreviations

ACT: Acceptance and Commitment Therapy
AIS: Avoidance and Inflexibility Scale
OR: odds ratio
PPA: point-prevalence abstinence
RCT: randomized controlled trial
USCPG: US Clinical Practice Guidelines
VQ: Valuing Questionnaire
