# Supplementary figures and images for: Mechanisms of Smartphone Apps for Cigarette Smoking Cessation: Results of a Serial Mediation Model From the iCanQuit Randomized Trial
Source: JMIR Mhealth Uhealth. 2021 Nov 9;9(11):e32847. doi: 10.2196/32847 (PMC8663588; doi:10.2196/32847)

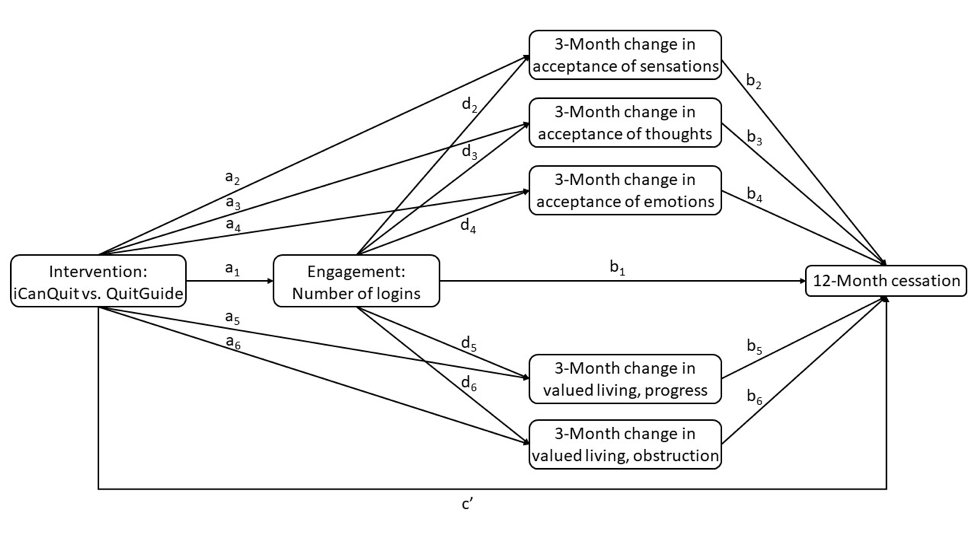

Supplement: Multimedia Appendix 1 [file mhealth_v9i11e32847_app1.png]
