# Supplementary material for: Mechanisms of Smartphone Apps for Cigarette Smoking Cessation: Results of a Serial Mediation Model From the iCanQuit Randomized Trial
Source: JMIR Mhealth Uhealth. 2021 Nov 9;9(11):e32847. doi: 10.2196/32847 (PMC8663588; doi:10.2196/32847)
Supplement: Multimedia Appendix 2 [file mhealth_v9i11e32847_app2.docx]

**Supplementary Table.** Estimates of indirect effects for pathways in a serial mediation model including the three AIS subscales.

| Mediator | Path | Estimate of the indirect effect (95% CI) for complete-case cessation outcome |
| --- | --- | --- |
| Number of logins | $a_{1}b_{1}$ | 0.09 (0.04, 0.17) |
| Change in acceptance of physical cues to smoke | $a_{2}b_{2}$ | 0.03 (0.002, 0.06) |
| Change in acceptance of thought cues to smoke | $a_{3}b_{3}$ | 0.01 (-0.02, 0.04) |
| Change in acceptance of emotional cues to smoke | $a_{4}b_{4}$ | 0.09 (0.03, 0.16) |
| Change in valued living, progress subscale | $a_{5}b_{5}$ | 0.00 (-0.02, 0.01) |
| Change in valued living, obstruction subscale | $a_{6}b_{6}$ | 0.00 (-0.01, 0.01) |
| Number of logins and change in acceptance of physical cues, in serial | $a_{1}{d_{2}b}_{2}$ | 0.03 (0.01, 0.05) |
| Number of logins and change in acceptance of thought cues, in serial | $a_{1}{d_{3}b}_{3}$ | 0.01 (-0.01, 0.03) |
| Number of logins and change in acceptance of emotional cues, in serial | $a_{1}{d_{4}b}_{4}$ | 0.07 (0.04, 0.11) |
| Number of logins and change in valued living progress, in serial | $a_{1}{d_{5}b}_{5}$ | 0.00 (-0.001, 0.01) |
| Number of logins and change in valued living obstruction, in serial | $a_{1}{d_{6}b}_{6}$ | 0.00 (-0.003, 0.002) |
